# Recurrence after preoperative chemotherapy and surgery for gastric adenocarcinoma: a multicenter study

**DOI:** 10.1007/s10120-019-00956-6

**Published:** 2019-04-04

**Authors:** I. Mokadem, W. P. M. Dijksterhuis, M. van Putten, L. Heuthorst, J. M. de Vos-Geelen, N. Haj Mohammad, G. A. P. Nieuwenhuijzen, H. W. M. van Laarhoven, R. H. A. Verhoeven

**Affiliations:** 1 Department of Research, Netherlands Comprehensive Cancer Organization (IKNL), PO Box 19079, 3501 DB Utrecht, The Netherlands; 2 Department of Medical Oncology, Cancer Center Amsterdam, Amsterdam UMC, University of Amsterdam, Amsterdam, The Netherlands; 3 Department of Surgery, Radboud University Medical Center, Nijmegen, The Netherlands; 4 Division of Medical Oncology, Department of Internal Medicine, GROW-School for Oncology and Developmental Biology, Maastricht UMC+, Maastricht, The Netherlands; 5 Department of Medical Oncology, University Medical Center Utrecht, Utrecht University, Utrecht, The Netherlands; 6 Department of Surgery, Catharina Hospital, Eindhoven, The Netherlands

**Keywords:** Gastric neoplasms, Perioperative care, Drug therapy, Neoadjuvant therapy, Adjuvant chemotherapy, Recurrence

## Abstract

**Background:**

In most western European countries perioperative chemotherapy is a part of standard curative treatment for gastric cancer. Nevertheless, recurrence rates remain high after multimodality treatment. This study examines patterns of recurrence in patients receiving perioperative chemotherapy with surgery for gastric cancer in a real-world setting.

**Methods:**

All patients diagnosed with gastric adenocarcinoma between 2010 and 2015 who underwent at least preoperative chemotherapy and a gastrectomy with curative intent (cT1N+/cT2-4a,X; any cN; cM0) in 18 Dutch hospitals were selected from the Netherlands Cancer Registry. Additional data on chemotherapy and recurrence were collected from medical records. Rates, patterns, and timing of recurrence were examined. Multivariable Cox proportional hazard analyses were used to determine prognostic factors for recurrence.

**Results:**

408 patients were identified. After a median follow-up of 27.8 months, 36.8% of the gastric cancer patients had a recurrence of which the majority (88.8%) had distant metastasis. The 1-year recurrence-free survival was 71.8%. The risk of recurrence was higher in patients with an ypN+ stage (HR 4.92, 95% CI 3.35–7.24), partial or no tumor regression (HR 2.63, 95% CI 1.22–5.64), 3 instead of ≥ 6 chemotherapy cycles (HR 3.04, 95% CI 1.99–4.63), R1 resection (HR 1.52, 95% CI 1.02–2.26), and < 15 resected lymph nodes (HR 1.64, 95% CI 1.14–2.37).

**Conclusion:**

A considerable amount of gastric cancer patients who were treated with curative intent developed a recurrence despite surgery and perioperative treatment. The majority developed distant metastases, therefore, multimodality treatment approaches should be focused on the prevention of distant rather than locoregional recurrences to improve survival.

## Introduction

Gastric cancer is the fourth most common malignancy and the second leading cause of cancer death worldwide [1, 2]. There is a strong geographical variation in the incidence of gastric cancer, with higher rates observed in Asian countries [2]. Over the past century, the incidence declined steadily in Europe, however, with a 5-year relative survival rate of 18–33%, survival remains poor [3].

Therefore, over the last 2 decades several studies have focused on different perioperative and postoperative multimodality approaches, to eliminate possible residual tumor and micrometastasis to reduce the risk of recurrence [4–10]. In the MAGIC trial, perioperative chemotherapy showed an improvement in 5-year overall survival compared to surgery alone (36% vs. 23%, respectively) [5]. However, only 42% of the patients randomized in the perioperative chemotherapy arm were able to complete the full treatment regimen due to early disease progression, toxicity, lack of response to preoperative treatment, and postoperative complications. Similar percentages were found in studies with other perioperative treatment regimens in gastric cancer [9, 10].

Previous studies demonstrated that, despite multimodality treatment approaches, recurrence occurs in 14–60% of patients and is one of the main factors associated with death in patients with gastric cancer [11–17]. The majority of recurrences occur in the first 2 years after resection and are frequently distant (42–47%) rather than locoregionally (16–34%) [12–15]. Studies that have examined recurrence of gastric cancer were mostly single institution studies in Asia [6, 14, 16–18]. Asian countries have a higher overall incidence rate of gastric cancer, a higher incidence rate of intestinal-type tumors and a different distribution of risk factors compared to Europe [2]. Moreover, guidelines in the Netherlands and several other European countries nowadays recommend perioperative chemotherapy with a (partial) gastrectomy as curative treatment of gastric cancer, while guidelines in the US and Asia recommend postoperative chemo(radio)therapy alone [4, 19, 20]. These differences in tumor, population and perioperative treatment characteristics together probably result in different survival rates, although more factors should be explored [21]. Because of these differences in populations and treatment of gastric cancer, information on rates and patterns of recurrence derived from a European population is lacking. Therefore, the aim of this study is to determine the recurrence-free survival and patterns of recurrence in patients receiving pre- or perioperative chemotherapy with a gastrectomy for gastric cancer. Furthermore, we aim to identify prognostic factors of gastric cancer recurrence.

## Methods

### Study population

All patients diagnosed with gastric adenocarcinoma between 2010 and 2015 who underwent at least preoperative chemotherapy and a gastrectomy with curative intent (cT1N+/cT2-4a,X; any cN; cM0) in 18 Dutch hospitals were included. These hospitals covered the whole Southeastern and Eastern part of the Netherlands and two hospitals in Amsterdam and Utrecht, including four academic hospitals, 12 teaching hospitals and two non-teaching hospitals, and can, therefore, be considered as adequately reflecting the nationwide patient population and hospitals. A resection was considered to be performed with curative intent if patients had no tumor infiltrating into surrounding organs (no cT4b) and/or no clinical distant metastasis (no cM1). All included patients were treated with either a (sub-)total gastrectomy or multi-organ resection, defined as a gastrectomy in combination with surgical removal of other organs. According to the Dutch gastric cancer guideline, patients were not eligible for perioperative chemotherapy if they had a cT1N0 tumor [19]. Patients with a cardia tumor were excluded if they were treated with preoperative chemoradiotherapy and an esophagectomy according to the guideline for esophageal cancer. To obtain a homogeneous study population of patients that received chemotherapy and a resection, we included all patients who completed at least one cycle of preoperative chemotherapy and underwent a gastric resection of the primary tumor.

### Data collection

Data were obtained from the Netherlands Cancer Registry (NCR), which is a population-based registry that includes all newly diagnosed malignancies in the Netherlands. The NCR contains data on diagnosis and treatment that are prospectively collected from medical records by specially trained data managers after notification by the national automated pathological archive. Annual linkage of the NCR to the Municipal Administrative Database has provided information on vital status and emigration of patients until 1 February 2017. This study was approved by the Privacy Review Board of the Netherlands Cancer Registry and the scientific committee of the Dutch Upper GI Cancer Group, and after official application did not require approval from an ethics committee in the Netherlands.

In the NCR, morphology and anatomical site of the tumor were registered according to the International Classification of Disease-Oncology (ICD-O-3). Anatomical site of the gastric tumor was categorized as follows: cardia (C16.0), proximal/middle (fundus, corpus, and lesser/greater curvature, (C16.1, C16.2, C16.5, C16.6), antrum (C16.3), pyloric (C16.4) and overlapping or not otherwise specified (C16.8, C16.9). Tumor staging was performed according to TNM-7 of the International Union Against Cancer (UICC) [22].

Since the NCR was collected retrospectively from medical records, some data were incomplete such as the ASA score, Lauren’s classification, Mandard tumor regression grade [23, 24], type of pre- and postoperative chemotherapy, number of received chemotherapy cycles, recurrence-related information, and date of last follow-up.

Patients were categorized as having a recurrence if their medical record described a histologically or FNA-proven recurrence or if they had imaging results indicating tumor recurrence. Recurrence patterns were classified as locoregional (locoregional lymph nodes, gastric or anastomotic tumor), distant peritoneal, or distant lymph node and/or hematogenous. In case the type of chemotherapy was changed during pre- or postoperative treatment—e.g., from ECC to EOX—patients were categorized as having received the type of chemotherapy that was given the most frequent (i.e., more cycles). Reasons for change and/or discontinuation of chemotherapy were not registered.

### Statistical analysis

Descriptive statistics were used to characterize the study population according to whether or not a patient developed a recurrence. Differences between groups were assessed by the Chi-square test, or the Fisher exact test when groups were smaller than five. The recurrence-free survival (RFS) was defined as the interval between the date of initial surgery and the date of (the first) recurrence, or death of any cause, or last follow-up, whichever occurred first. Follow-up for survival was available until the 1st of February 2017. The RFS was assessed using the Kaplan–Meier method and differences between groups were assessed by the log rank test. Uni- and multivariable Cox proportional hazard analyses were used to determine prognostic factors for the development of recurrences. Variables that caused multicollinearity were excluded from the multivariable analysis. Missing variables are displayed and included as a separate categorical variable in uni- and multivariable analyses. Results were reported as hazard ratios (HRs) with 95% confidence interval (CI). A *P* value of < 0.05 was considered statistically significant. SAS version 9.4 (Statistical Analysis System) was used for all analyses.

## Results

### Patients

Between 2010 and 2015, 408 patients underwent at least 1 cycle of preoperative chemotherapy and a gastrectomy with curative intent in the 18 selected hospitals. Patient and tumor characteristics are shown in Table 1. Median age of all patients was 66 years [interquartile range (IQR) 57, 71] and 64.2% was male. Among the patients with a registered type of chemotherapy, the majority of the patients (90.7%) received preoperative chemotherapy containing epirubicin and cisplatin in combination with capecitabine or fluorouracil (ECX/ECF; 62.0%), or epirubicin and oxaliplatin in combination with capecitabine or fluorouracil (EOX/EOF; 28.7%). During preoperative chemotherapy the type of chemotherapy was changed in seven patients, five switched from ECX/ECF to EOX/EOF, and two from EOX/EOF to ECX/ECF. Of the 322 (78.9%) cases in which the number of preoperative cycles was known, 56 (17.4%) patients received less than 3 cycles of preoperative chemotherapy and, therefore, did not complete the full preoperative treatment regimen. Postoperative therapy was administered in 266 (65.3%) patients, 205 (77.1%) of these patients received postoperative chemotherapy and the other patients received chemoradiotherapy according to the CRITICS trial protocol [25]. Of patients that started postoperative chemotherapy, in 149 (72.2%) patients the number of cycles was known. Of these 149 patients, postoperative chemotherapy was not completed in 37 (24.8%) patients and completed in 112 (74.2%) patients. Complete [ypT0; tumor regression grade (TRG) 1], subtotal (TRG 2) partial (TRG 3–4) or no tumor regression (TRG 5) were present in 5.9, 4.7, 20.8, and 12.8% of the patients, respectively, however, it was unknown in 55.9% of the patients.

**Table 1:** Patient and tumor characteristics in terms of recurrence for gastric cancer patients diagnosed in 2010–2015 and treated with curative intent (*N* = 408)

Characteristics	All patients		Recurrence		No recurrence		*P* value
	*N*	%^a^	*N*	%^b^	*N*	%^b^	
	408	100	150	36.8	258	63.2	
Age							0.1107
≥ 70 years	143	35.1	43	30.1	100	69.9	
60–69 years	145	35.5	57	39.3	88	60.7	
< 60 years	120	29.4	50	41.7	70	58.3	
Sex							0.6187
Male	262	64.2	94	35.9	168	64.1	
Female	146	35.8	56	38.4	90	61.6	
Comorbidities							< 0.0001
None	100	24.51	52	52.0	48	48.0	
1	99	24.26	45	45.5	54	54.5	
2 or more	100	24.51	41	41.0	59	59.0	
Unknown	109	26.72	12	11.0	97	89.0	
Type of preoperative chemotherapy							0.2389
Triplet chemotherapy	311	76.2	118	37.9	193	62.1	
*ECX/ECF*	*201*	*49.3*	*79*	*39.3*	*122*	*60.7*	
*EOX/EOF*	*93*	*22.8*	*34*	*36.6*	*59*	*63.4*	
*DOCCS*	*16*	*3.9*	*4*	*25.0*	*12*	*75.0*	
*Other triplet chemotherapy*	*1*	*0.3*	*1*	*100.0*	*0*	*0.0*	
Doublet chemotherapy	12	2.9	1	0.7	11	4.3	
*CAP*-*/FOLFOX*	*11*	*2.7*	*1*	*9.1*	*10*	*90.9*	
*Carboplatin *+* paclitaxel*	*1*	*0.3*	*0*	*0.0*	*1*	*100.0*	
Mono chemotherapy	1	0.3	1	100.0	0	0.0	
Unknown	84	20.6	30	35.7	54	64.3	
Postoperative therapy							0.0828
None	142	34.8	47	33.1	95	66.9	
Chemoradiotherapy	61	15.0	30	49.2	31	50.8	
Chemotherapy	205	50.3	73	35.6	132	64.4	
*ECX/ECF*	*93*	*45.4*	*34*	*36.6*	*59*	*63.4*	
*EOX/EOF*	*48*	*23.4*	*17*	*35.4*	*31*	*35.4*	
*Other chemotherapy*	*64*	*31.2*	*22*	*34.4*	*42*	*60.7*	
Number of chemotherapy cycles							0.8693
1 or 2	53	13.0	20	37.7	33	62.3	
3	106	26.0	40	37.7	66	62.3	
4 or 5	48	11.8	20	41.7	28	58.3	
6 or more	115	28.2	38	33.0	77	77.0	
Unknown	86	21.1	32	37.2	54	62.8	
ASA score							0.0382
Class I	38	9.3	11	28.9	27	71.1	
Class II	241	59.1	87	36.1	154	63.9	
Class III	72	17.7	22	30.6	50	69.4	
Unknown	57	14.0	30	52.6	27	47.4	
Lauren’s classification							0.0071
Diffuse	189	46.3	86	45.5	103	54.5	
Intestinal	154	37.8	43	27.9	111	72.1	
Mixed	35	8.6	12	34.3	23	65.7	
Unknown	30	7.4	9	30.0	21	70.0	
Tumor regression							0.0004
Complete	24	5.9	3	12.5	21	87.5	
Subtotal	19	4.7	4	21.0	15	79.0	
Partial	85	20.8	33	38.8	52	61.2	
None	52	12.8	31	59.6	21	40.4	
Unknown	228	55.9	79	34.7	149	65.4	
ypT stage							< 0.0001
T0	30	7.4	3	10.0	27	90.0	
T1	52	12.8	6	11.5	46	88.5	
T2	59	14.5	12	20.3	47	79.7	
T3	175	42.9	78	44.6	97	55.4	
T4	90	22.1	51	56.7	39	43.3	
TX	2	0.5	0	0.0	2	100.0	
ypN stage							< 0.0001
N0	185	45.3	31	16.8	154	83.2	
N+	223	54.7	119	53.4	104	46.6	
Resected lymph nodes							0.3550
< 15	89	21.81	29	32.6	60	67.4	
15 or more	319	78.19	121	37.9	198	62.1	
Differentiation grade							0.0805
Well/moderately	52	12.8	13	25.0	39	75.0	
Poorly/undifferentiated	204	50.0	84	41.2	120	58.8	
Unknown	152	37.3	53	34.9	99	65.1	
Resection margin							< 0.0001
R0	346	84.8	112	32.4	234	67.6	
R1/R2	48	11.8	30	62.5	18	37.5	
Unknown	14	3.4	8	57.1	6	42.9	
Tumor location							0.0054
Cardia	28	6.9	9	32.1	19	67.9	
Proximal/middle	150	36.8	54	36.0	96	64.0	
Antrum	110	27.0	28	25.5	82	74.5	
Pyloric	23	5.6	10	43.5	13	56.5	
Other	97	23.8	49	50.5	48	49.5	

*Cursive*: distribution of the type of chemotherapy

*ECX/ECF* epirubicin + cisplatin + capecitabine or 5-fluorouracil, *EOX/EOF* epirubicin + oxaliplatin + capecitabine or 5-fluorouracil, *DOCCS* docetaxel + cisplatin + capecitabine, *CAP-/FOLFOX* capecitabine or 5-fluorouracil + oxaliplatin, *CT* chemotherapy, *ASA* American Society of Anesthesiology

^a^Column percentages

^b^Row percentages

### Follow-up and recurrence rates

After a median follow-up time of 27.8 months (range 0.1–83.2 moths), 175 patients (42.9%) had died at the end of follow-up. Recurrence was diagnosed in 36.8% of the patients (Table 1). No difference in the proportion of recurrence was observed for age, sex, type of preoperative chemotherapy, number of cycles of chemotherapy, and differentiation grade. Recurrence was less often present in patients with ASA I-score (*P* = 0.040), a tumor in the antrum (*P* = 0.005), an intestinal tumor type (*P* = 0.007), an ypT0 tumor (*P* < 0.001), an ypN0 tumor (*P* < 0.001), with complete pathological regression (ypT0) after preoperative chemotherapy (*P* = 0.001), and R0 resection (*P* < 0.001).

### Patterns of recurrence

**Fig. 1:**
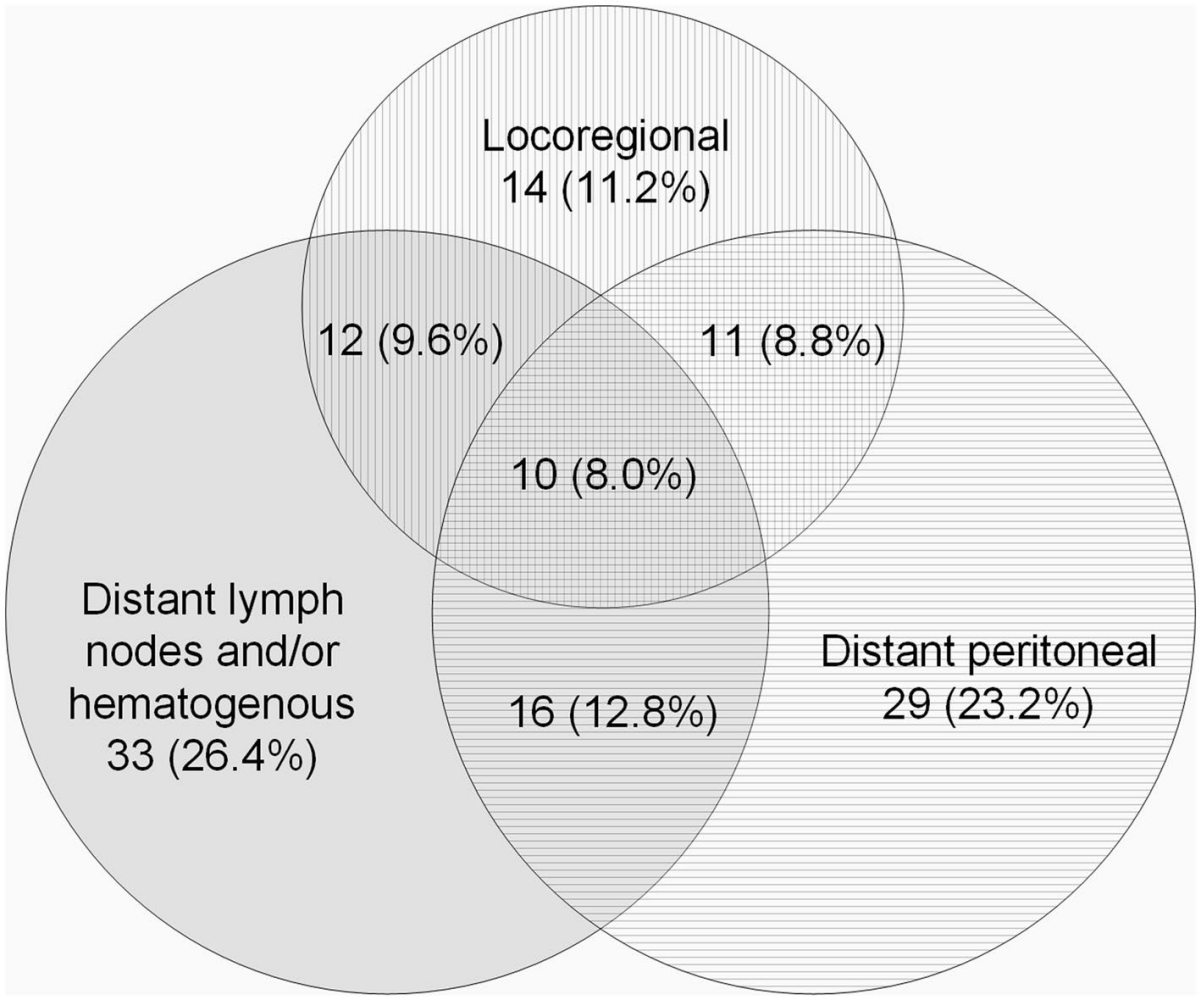
Overall pattern of recurrence of gastric cancer patients diagnosed with a recurrence (*N* = 125)

Information on the location of the recurrence was available for 125 (83.3%) patients (Fig. 1). Overall, 76 patients (60.8%) had recurrence at a single site. Among patients who had recurrence at a single site, distant recurrences were most common (81.6%) including 29 patients (46.8%) with peritoneal and 33 patients (53.2%) with distant lymph node and/or hematogenous recurrences. Only 11.2% (*N* = 14) of the patients had locoregional recurrence without distant recurrence. Overall, locoregional recurrences were found in 47 patients (37.6%), distant peritoneal recurrences in 66 patients (52.8%), and distant lymph node and/or hematogenous recurrences in 71 patients (56.8%). The most prevalent sites of distant lymph node and/or hematogenous recurrence were liver (*N* = 33, 46.5%), lung (*N* = 11, 15.5%), and intra-thoracic lymph nodes (*N* = 15, 21.1%).

### Time interval until recurrence

Median RFS among all patients was 22.5 months (IQR 10.0, 42.3). The 1-, 2- and 3-year RFS rates were 71.8, 58.6, and 47.4%, respectively. Among patients with recurrence, median RFS was 10.8 months (IQR 6.7, 20.0). Median RFS was shorter in patients that had distant peritoneal recurrence (7.5 months; IQR 5.0, 11.0) compared to patients with distant lymph node and/or hematogenous recurrence (14.1 months; IQR 7.5, 21.6), or patients with locoregional recurrence only (14.3 months, IQR 8.6, 30.1). Among patients without recurrence, median OS was 32.7 months (IQR 18.1, 51.9).

### Multivariable analyses to identify factors associated with recurrence

**Fig. 2:**
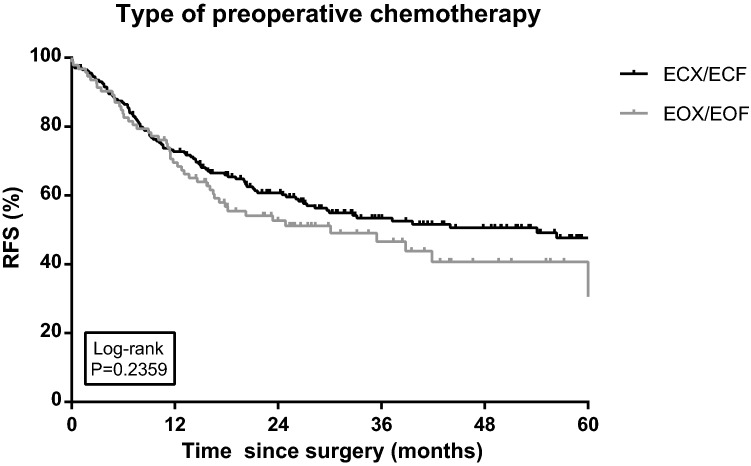
Recurrence-free survival (RFS) among gastric cancer patients diagnosed in 2010–2015 and treated with preoperative chemotherapy including epirubicin and cisplatin in combination with capecitabine or fluorouracil (ECX/ECF), or epirubicin and oxaliplatin in combination with capecitabine or 5-fluorouracil (EOX/EOF) (*N* = 201 and *N* = 93, respectively). RFS was defined as the interval between the date of initial operation and the date of (the first) recurrence, or death of any cause, or last follow-up (follow-up data available until 1 February 2017), whichever occurred first. Ticks represent censored patients

**Fig. 3:**
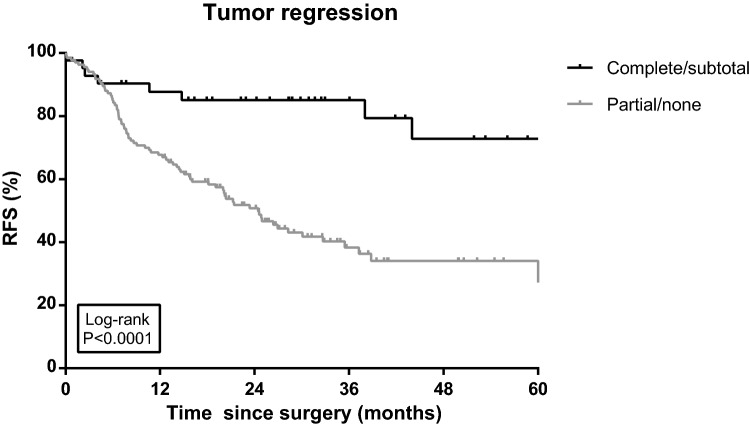
Recurrence-free survival (RFS) among gastric cancer patients diagnosed in 2010–2015 with complete/subtotal (*N* = 43) tumor regression or partial/no (*N* = 137) tumor regression after preoperative chemotherapy. RFS was defined as the interval between the date of initial operation and the date of (the first) recurrence, or death of any cause, or last follow-up (follow-up data available until 1 February 2017), whichever occurred first. Ticks represent censored patients

**Fig. 4:**
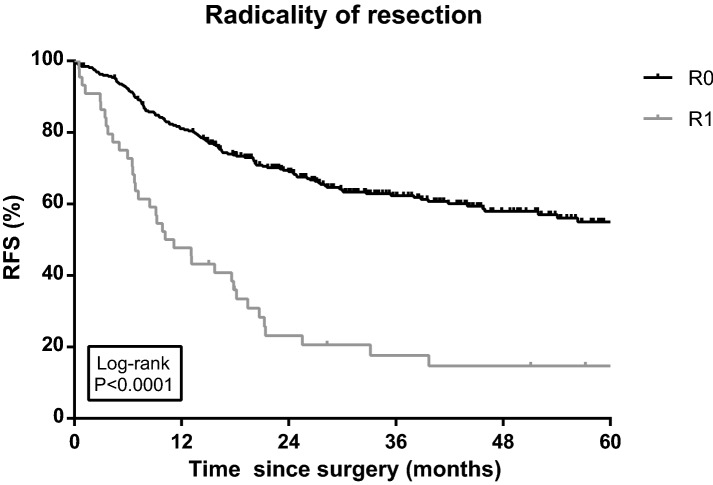
Recurrence-free survival (RFS) among gastric cancer patients diagnosed in 2010–2015 and treated with preoperative chemotherapy after R0 or R1 resection. RFS was defined as the interval between the date of initial operation and the date of (the first) recurrence, or death of any cause, or last follow-up (follow-up data available until 1 February 2017), whichever occurred first. Ticks represent censored patients

The type of preoperative chemotherapy was not associated with a difference in RFS in univariable analysis (Log rank *P* = 0.236; Fig. 2). Partial or no tumor regression and positive resection margins were associated with differences in RFS in both univariable (Log rank *P* < 0.001 < 0.001; Figs. 3 and 4) and multivariable Cox regression analyses ([HR 2.63, 95% CI 1.22–5.64) and (HR 1.52, 95% CI 1.02–2.26), respectively; Table 2]. Furthermore, three rather than six or more cycles of chemotherapy (including chemotherapy cycles of chemoradiation; HR 3.04, 95% CI 1.99–4.63), an advanced ypN stage (ypN+; HR 4.92, 95% CI 3.35–7.24), diffuse type tumors (HR 1.60, 95% CI 1.09–2.35) and less than 15 resected lymph nodes (HR 1.64, 95% CI 1.14–2.37) were independent prognostic factors for recurrence (Table 2). The risk of recurrence was significantly lower in patients aged below 60 (HR 0.64, 95% CI 0.42–0.97).

**Table 2:** Recurrence-free survival of gastric cancer patients diagnosed in 2010–2015 and treated with curative intent (*N* = 408)

Characteristics	Events^a^	Univariable				Multivariable			
		HR	95% CI		*P* value	HR	95% CI		*P* value
			Lower	Upper			Lower	Upper	
Age									
≥ 70 years	70	Ref				Ref			
60–69 years	70	0.95	0.68	1.32	0.740	1.01	0.70	1.45	0.965
< 60 years	58	0.93	0.66	1.32	0.684	***0.64***	***0.42***	***0.97***	***0.034***
Sex									
Male	128	Ref				Ref			
Female	70	0.99	0.74	1.32	0.923	1.04	0.75	1.44	0.811
Comorbidities									
None	56	Ref				–			
1	53	0.95	0.65	1.38	0.780	–			
2 or more	62	1.33	0.92	1.91	0.126	–			
Unknown	27	0.36	0.23	0.57	**< ** ***0.001***	–			
Type of preoperative chemotherapy									
Triplet chemotherapy									
ECX/ECF	94	Ref				Ref			
EOX/EOF	48	1.24	0.87	1.75	0.233	1.26	0.86	1.85	0.242
Other triplet chemotherapy	8	0.86	0.42	1.78	0.692	1.20	0.53	2.73	0.664
Mono/doublet chemotherapy	8	***2.07***	***1.00***	***4.26***	***0.049***	1.62	0.73	3.60	0.241
Unknown	40	0.94	0.65	1.36	0.122	1.64	0.26	10.40	0.599
Number of chemotherapy cycles									
1 or 2	53	1.43	0.88	2.33	0.154	1.48	0.87	2.54	0.152
3	106	***2.04***	***1.39***	***2.99***	**< ** ***0.001***	***3.04***	***1.99***	***4.63***	**< ** ***0.001***
4 or 5	48	1.21	0.73	2.00	0.459	1.16	0.67	2.02	0.602
6 or more	115	Ref				Ref			
Unknown	86	1.21	0.79	1.84	0.383	0.88	0.14	5.41	0.891
Adjuvant (postoperative) treatment									
No adjuvant treatment	142	***1.76***	***1.29***	***2.39***	**< ** ***0.001***	–			
Chemotherapy	205	Ref				–			
Chemoradiation	61	1.44	0.97	2.14	0.073	–			
ASA score									
Class I	12	0.59	0.32	1.06	0.078	0.73	0.39	1.38	0.332
Class II	112	Ref				Ref			
Class III	37	1.25	0.86	1.81	0.246	1.13	0.75	1.71	0.566
Unknown	37	***1.75***	***1.20***	***2.54***	***0.003***	***2.32***	***1.51***	***3.57***	**< ** ***0.001***
Lauren’s classification									
Intestinal	106	Ref				Ref			
Diffuse	60	***1.65***	***1.20***	***2.26***	***0.002***	***1.60***	***1.09***	***2.35***	***0.018***
Mixed	17	1.25	0.73	2.14	0.417	1.03	0.58	1.85	0.914
Unknown	15	1.57	0.89	2.77	0.117	1.57	0.84	2.91	0.156
Tumor regression									
Complete/subtotal	8	Ref				Ref			
Partial/none	81	***4.06***	***1.96***	***8.40***	**< ** ***0.001***	***2.63***	***1.22***	***5.64***	***0.013***
Unknown	109	***2.64***	***1.29***	***5.40***	***0.008***	2.08	0.96	4.50	0.062
ypT stadium									
T0–2, X	31	Ref				–			
T3	98	***3.32***	***2.21***	***4.98***	**< ** ***0.001***	–			
T4	69	***6.07***	***3.96***	***9.31***	**< ** ***0.001***	–			
ypN stadium									
N0	46	Ref				Ref			
N+	152	***4.38***	***3.13***	***6.12***	**< ** ***0.001***	***4.92***	***3.35***	***7.24***	**< ** ***0.001***
Number of resected lymph nodes									
< 15	47	1.08	0.78	1.50	0.651	***1.64***	***1.14***	***2.37***	***0.008***
15 or more	151	Ref				Ref			
Resection margin									
R0	147	Ref				Ref			
R1/R2	40	***3.10***	***2.18***	***4.40***	**< ** ***0.001***	***1.52***	***1.02***	***2.26***	***0.041***
Unknown	11	***3.61***	***1.95***	***6.69***	**< ** ***0.001***	1.73	0.88	3.40	0.110
Differentiation grade									
Well/moderately	19	***0.60***	***0.37***	***0.98***	***0.042***	0.67	0.39	1.17	0.156
Poorly/undifferentiated	109	Ref				Ref			
Unknown	70	0.79	0.58	1.06	0.117	0.83	0.60	1.14	0.250
Tumor location									
Cardia	14	1.22	0.69	2.17	0.500	1.27	0.67	2.40	0.464
Proximal/middle	69	Ref				Ref			
Antrum	41	0.79	0.53	1.16	0.220	0.74	0.48	1.14	0.177
Pyloric	12	1.22	0.66	2.26	0.523	0.86	0.44	1.69	0.662
Other	62	***1.69***	***1.20***	***2.38***	***0.003***	1.41	0.96	2.06	0.079

Comorbidities, adjuvant (postoperative) treatment and ypT stadium were not included in multivariable analysis to avoid multicollinearity

*HR* hazard ratio, *CI* confidence interval, *ECX/ECF* epirubicin + cisplatin + capecitabine or 5-fluorouracil, *EOX/EOF* epirubicin + oxaliplatin + capecitabine or 5-fluorouracil, *ASA* American Society of Anesthesiology

*Cursive* and **bold**: values are statistically significant, *P* < 0.05

^a^Event: recurrence (local and/or distant) or death, whichever occurred first

## Discussion

This multicenter study demonstrated that after a median follow-up of 27.8 months, more than a third of the gastric cancer patients who were treated with at least one cycle of preoperative chemotherapy and a gastrectomy with curative intent developed a recurrence, of which the far majority (90%) developed a distant metastasis. This study identified age above 60, R1 resection margin, ypN+ tumor stage, diffuse type tumors, partial or no tumor regression, and no postoperative therapy as independent associated factors with a higher chance on recurrence.

Rates of recurrence in gastric cancer patients vary from 14–60% within the current literature [11–17, 26]. Comparison of these results is difficult, because of different inclusion criteria, methodological variation and disparity in treatment regimens [11–14], and patient populations [15–17, 26]. Our results confirm previous studies that distant recurrences are the most common type of recurrence in gastric cancer patients after curative surgery [12, 13, 26]. For example, Spolverato and colleagues [12] reported that nearly three of every four patients who experienced a recurrence had at least a distant recurrence. Also, survival was worse after recurrence had occurred in patients diagnosed with distant recurrence compared to patients with locoregional recurrence. In line with previous findings, the present study revealed that 82% of patients who had a single site recurrence had a distant recurrence, and almost 90% of all with a recurrence showed a distant component (Fig. 1). Distant recurrences were mostly located in the peritoneum, liver, lungs and intra-thoracic lymph nodes. The dissemination to specific organs may partly be explained by drainage of tumor cells in the vascular and lymphatic system or shedding of tumor cells from the surface of the primary tumor into the peritoneal cavity [12].

Earlier studies have reported that the majority of recurrences occurs within 2–3 years after surgery which is in line with our findings [12, 26]. Adequate surveillance for recurrences, if any, is, therefore, particularly important within the first 3 years after surgery. Currently, there is no consensus on how follow-up should be performed or how frequently it should be scheduled [27]. The aim of follow-up is to diagnose recurrences in an early stage to enhance the possibility of curative treatment after a recurrence has been detected. However, an increase in overall survival in gastric cancer patients after an early diagnosis of recurrence has not been demonstrated in previous research [27], probably due to the fact that most recurrences are distant recurrences which are not amenable to curative treatment. It remains to be shown that early start of palliative systemic treatment and palliative care improves survival and quality of life.

Earlier studies found that patients with a Lauren diffuse-type tumor had a higher rate of recurrence compared to the intestinal type [13, 17], and that tumor regression was lowest in diffuse-type tumors [28]. Our study demonstrates that the risk of recurrence is higher in patients with partial or no pathological tumor regression and patients with diffuse-type tumors. Moreover, highest HR for recurrence was found in patients with an advanced ypN stage (ypN+; HR 4.9), which is in line with earlier studies [11, 15, 26].

Eventually, amendable factors which could substantially affect RFS are perioperative systemic and surgical treatment outcomes. Our results demonstrate that the type of preoperative chemotherapy did not predict the risk of recurrence. However, in the future this may change due to introduction of the FLOT (5-FU, folinic acid, oxaliplatin, docetaxel) regimen, which has appeared to be superior to ECF and ECX [9]. Nonetheless, recurrence was more frequent among patients that received only three cycles of chemotherapy in combination with surgery, compared to those who underwent six or more cycles of chemotherapy and surgery. In our description of ‘real world’ patterns of care only 28% of the patients completed the perioperative regimen, which is substantially lower than the 42% completion rate in the MAGIC trial [5]. Therefore, it may be argued that to improve treatment outcome, improved tolerability of systemic therapy rather than increased intensity should be the focus of further research. Given the lower tolerability of these regimens, at least in Western countries, one way to achieve this goal may be to focus more on neoadjuvant treatment regimens, rather than perioperative or adjuvant treatment, like in the recently started CRITICS II trial [29]. Reasons for not completing postoperative treatment have appeared to include high toxicity of preoperative chemotherapy, early disease progression, and postoperative complications [5]. Therefore, initiatives for adjusting perioperative treatment to only neoadjuvant treatment emerge, however, questions on the optimal number of cycles remain.

Besides systemic therapy, quality of surgical treatment may also influence the probability of recurrence, as positive resection margins and resection of less than 15 lymph nodes were independently associated with higher recurrence rates as well. Due to centralization of surgery in the Netherlands, the rate of R1 resections has already significantly decreased over the past few years [30]. However, an adequate lymphadenectomy, increased use of perioperative treatment and the standard use of, for example, intraoperative frozen section should remain an important concern to prevent locoregional recurrences. On the other hand, some possible causes of an R1 resection, such as aggressive tumor biology, are harder to influence than inadequate surgery techniques and perioperative treatment strategies. Moreover, recurrence rates appeared to be independently associated with ypN+ stage and, therefore, chances of recurrence remain significant in patients with pathologic-positive lymph nodes, even after R0 resection.

This study is not without limitations. First, some variables had a high rate of missing values, with the highest percentages for tumor regression, type of preoperative chemotherapy, number of preoperative chemotherapy cycles, and type of postoperative chemotherapy. These variables were missing for at least 84 patients, because their data were collected prior to this study for other purposes. However, we did have information on recurrence, last follow-up, ASA score and Lauren’s classification for these patients and, therefore, decided that is was useful to include these data. Second, we could not analyze which factors were associated with specific recurrence patterns. Previous studies that explored recurrence patterns found that different clinical pathological factors contributed to specific recurrence patterns [12, 13, 15, 17, 26]. Unfortunately, the small numbers of patients per pattern in our population precluded the investigation of prognostic factors for specific recurrence patterns. Last, the NCR did not have nationwide information WHO performance status. However, we did include ASA score, as a proxy parameter of performance status, which has shown to be an important predictor for morbidity and mortality after a gastrectomy [31].

A strength of this study is the use of the national cancer registry data and additionally collected data that provide information on recurrence, the type of chemotherapy and the completion of treatment, which are often missing in previous research. Furthermore, the present study is the first multicenter study that investigates the RFS in patients receiving preoperative chemotherapy with surgery for gastric cancer. This study has the advantage of including patients diagnosed in recent years from both academic hospitals and (non-)teaching hospitals, and, therefore, provides a good reflection of current clinical practice in The Netherlands.

In conclusion, this study provides an overview on patterns, timing, and prognostic factors for recurrence after curative treatment of gastric cancer patients. Despite perioperative treatment, a considerable number of patients who were treated with curative intent developed a recurrence during clinical follow-up. Multimodality treatment approaches to prevent recurrences in gastric cancer patients should be focused on the prevention of distant rather than locoregional recurrences.

## References

[1] Crew KD, Neugut AI (2006). Epidemiology of gastric cancer. World J Gastroenterol.

[2] Jemal A, Bray F, Center MM, Ferlay J, Ward E, Forman D (2011). Global cancer statistics. CA Cancer J Clin.

[3] Allemani C, Weir HK, Carreira H, Harewood R, Spika D, Wang XS (2015). Global surveillance of cancer survival 1995–2009: analysis of individual data for 25,676,887 patients from 279 population-based registries in 67 countries (CONCORD-2). Lancet.

[4] Macdonald JS, Smalley SR, Benedetti J, Hundahl SA, Estes NC, Stemmermann GN (2001). Chemoradiotherapy after surgery compared with surgery alone for adenocarcinoma of the stomach or gastroesophageal junction. N Engl J Med.

[5] Cunningham D, Allum WH, Stenning SP, Thompson JN, Van de Velde CJ, Nicolson M (2006). Perioperative chemotherapy versus surgery alone for resectable gastroesophageal cancer. N Engl J Med.

[6] Chiu CF, Yang HR, Yang MD, Jeng LB, Sargeant AM, Yeh SP (2016). The role of adjuvant chemotherapy for patients with stage II and stage III gastric adenocarcinoma after surgery plus D2 lymph node dissection: a real-world observation. SpringerPlus.

[7] Ychou M, Boige V, Pignon JP, Conroy T, Bouche O, Lebreton G (2011). Perioperative chemotherapy compared with surgery alone for resectable gastroesophageal adenocarcinoma: an FNCLCC and FFCD multicenter phase III trial. J Clin Oncol.

[8] Yang YN, Yin X, Sheng L, Xu S, Dong LL, Liu L (2015). Perioperative chemotherapy more of a benefit for overall survival than adjuvant chemotherapy for operable gastric cancer: an updated meta-analysis. Sci Rep.

[9] Al-Batran S-E, Homann N, Schmalenberg H, Kopp H-G, Haag GM, Luley KB (2017). Perioperative chemotherapy with docetaxel, oxaliplatin, and fluorouracil/leucovorin (FLOT) versus epirubicin, cisplatin, and fluorouracil or capecitabine (ECF/ECX) for resectable gastric or gastroesophageal junction (GEJ) adenocarcinoma (FLOT4-AIO): a multicenter, randomized phase 3 trial. J Clin Oncol.

[10] Cats A, Jansen EPM, van Grieken NCT, Sikorska K, Lind P, Nordsmark M (2018). Chemotherapy versus chemoradiotherapy after surgery and preoperative chemotherapy for resectable gastric cancer (CRITICS): an international, open-label, randomised phase 3 trial. Lancet Oncol.

[11] Seyfried F, von Rahden BH, Miras AD, Gasser M, Maeder U, Kunzmann V (2015). Incidence, time course and independent risk factors for metachronous peritoneal carcinomatosis of gastric origin—a longitudinal experience from a prospectively collected database of 1108 patients. BMC Cancer.

[12] Spolverato G, Ejaz A, Kim Y, Squires MH, Poultsides GA, Fields RC (2014). Rates and patterns of recurrence after curative intent resection for gastric cancer: a United States multi-institutional analysis. J Am Coll Surg.

[13] D’Angelica M, Gonen M, Brennan MF, Turnbull AD, Bains M, Karpeh MS (2004). Patterns of initial recurrence in completely resected gastric adenocarcinoma. Ann Surg.

[14] Chou HH, Kuo CJ, Hsu JT, Chen TH, Lin CJ, Tseng JH (2013). Clinicopathologic study of node-negative advanced gastric cancer and analysis of factors predicting its recurrence and prognosis. Am J Surg.

[15] Liu D, Lu M, Li J, Yang Z, Feng Q, Zhou M (2016). The patterns and timing of recurrence after curative resection for gastric cancer in China. World J Surg Oncol.

[16] Lee KW, Bang SM, Kim S, Lee HJ, Shin DY, Koh Y (2010). The incidence, risk factors and prognostic implications of venous thromboembolism in patients with gastric cancer. J Thromb Haemost.

[17] Huang KH, Chen JH, Wu CW, Lo SS, Hsieh MC, Li AF (2009). Factors affecting recurrence in node-negative advanced gastric cancer. J Gastroenterol Hepatol.

[18] Cheng J, Wu J, Ye Y, Zhang C, Zhang Y, Wang Y (2016). The prognostic significance of extramural venous invasion detected by multiple-row detector computed tomography in stage III gastric cancer. Abdom Radiol (NY).

[19] Oncoline. National clinical practice guideline gastric cancer. 2017. http://www.oncoline.nl/maagcarcinoom. Accessed 19 Nov 2018.

[20] Japanese Gastric Cancer Association (2011). Japanese gastric cancer treatment guidelines 2010 (ver 3). Gastric Cancer.

[21] Bickenbach K, Strong VE (2012). Comparisons of gastric cancer treatments: east vs. west. J Gastric Cancer.

[22] UICC (2009). TNM classification of malignant tumours.

[23] Mandard A-M, Dalibard F, Mandard J-C, Marnay J, Henry-Amar M, Petiot J-F (1994). Pathologic assessment of tumor regression after preoperative chemoradiotherapy of esophageal carcinoma. Clinicopathologic Correl. Cancer..

[24] Zhu Y, Sun Y, Hu S, Jiang Y, Yue J, Xue X (2017). Comparison of five tumor regression grading systems for gastric adenocarcinoma after neoadjuvant chemotherapy: a retrospective study of 192 cases from National Cancer Center in China. BMC Gastroenterol.

[25] Dikken JL, van Sandick JW, Maurits Swellengrebel HA, Lind PA, Putter H, Jansen EP (2011). Neo-adjuvant chemotherapy followed by surgery and chemotherapy or by surgery and chemoradiotherapy for patients with resectable gastric cancer (CRITICS). BMC Cancer.

[26] Wu CW, Lo SS, Shen KH, Hsieh MC, Chen JH, Chiang JH (2003). Incidence and factors associated with recurrence patterns after intended curative surgery for gastric cancer. World J Surg.

[27] Aurello P, Petrucciani N, Antolino L, Giulitti D, D’Angelo F, Ramacciato G (2017). Follow-up after curative resection for gastric cancer: is it time to tailor it?. World J Gastroenterol.

[28] Neves Filho EH, de Sant’Ana RO, Nunes LV, Pires AP, da Cunha MD (2017). Histopathological regression of gastric adenocarcinoma after neoadjuvant therapy: a critical review. APMIS.

[29] Slagter AE, Jansen EPM, van Laarhoven HWM, van Sandick JW, van Grieken NCT, Sikorska K (2018). CRITICS-II: a multicentre randomised phase II trial of neo-adjuvant chemotherapy followed by surgery versus neo-adjuvant chemotherapy and subsequent chemoradiotherapy followed by surgery versus neo-adjuvant chemoradiotherapy followed by surgery in resectable gastric cancer. BMC Cancer.

[30] van Putten M, Nelen SD, Lemmens V, Stoot J, Hartgrink HH, Gisbertz SS (2018). Overall survival before and after centralization of gastric cancer surgery in the Netherlands. Br J Surg.

[31] Nelen SD, Bosscha K, Lemmens V, Hartgrink HH, Verhoeven RHA, de Wilt JHW (2018). Morbidity and mortality according to age following gastrectomy for gastric cancer. Br J Surg.

